# Clinical Characteristics and Prognosis of Neuroendocrine Carcinoma in the Head and Neck: A Single-Institutional Retrospective Analysis

**DOI:** 10.3390/curroncol33070390

**Published:** 2026-06-29

**Authors:** Chengyan Yang, Kun Gao, Shuangshuang He, Mengyuan Liu, Ping Ai

**Affiliations:** 1 Division of Head & Neck Tumor Multimodality Treatment, Cancer Center, West China Hospital, Sichuan University, No. 37 Guoxue Alley, Chengdu 610041, China; 2 Department of Oncology, The Third Affiliated Hospital of Chongqing Medical University, Chongqing 401120, China

**Keywords:** head and neck neuroendocrine carcinoma, radiotherapy, prognosis, larynx

## Abstract

Background: Head and neck neuroendocrine carcinoma (HN-NEC) is exceedingly rare. Standardized treatment strategies for this malignancy remain unestablished. This study aimed to explore promising treatment modalities, and to identify prognostic factors in HN-NEC. Materials and Methods: Thirty-nine patients diagnosed with HN-NEC at West China Hospital of Sichuan University between 2006 and 2025 were enrolled. The 5-year survival rates were estimated by Kaplan–Meier analysis. The log-rank test and Firth’s penalized Cox multivariable analysis regression model were used to identify prognostic factors. Results: The 5-year locoregional recurrence-free survival (LRRFS), distant metastasis-free survival (DMFS), and overall survival (OS) rates for patients who did and did not receive radiotherapy were 63.2% vs. 29.6% (*p* = 0.031), 75.5% vs. 48.0% (*p* = 0.065), and 81.4% vs. 46.9% (*p* = 0.039), respectively. Laryngeal NEC was associated with poorer 5-year DMFS (41.2% vs. 87.5%, *p* = 0.023) and 5-year OS (38.1% vs. 92.9%, *p* = 0.027) compared with non-laryngeal HN-NEC. Radiotherapy (HR = 0.152, 95% CI: 0.025–0.757, *p* = 0.022) was a potentially protective factor influencing LRRFS. Conclusions: Radiotherapy may be associated with improved LRRFS in patients with HN-NEC. HN-NEC originating in the larynx appeared to be associated with a poorer prognosis compared with other primary sites of the head and neck.

## Simple Summary

Head and neck neuroendocrine carcinoma (HN-NEC) is a rare tumor with limited clinical data. Due to its rarity, large studies are lacking, and treatment decisions are often challenging. In this retrospective study of 39 patients, we analyzed the clinical features, treatment approaches, and survival outcomes of this rare tumor. Our findings suggest that radiotherapy is associated with achieving local control in HN-NEC. By sharing our single-center experience, we aim to contribute valuable data to the limited body of knowledge on this rare tumor.

## 1. Introduction

Neuroendocrine neoplasms (NENs) are a heterogeneous group of tumors with neuroendocrine differentiation. They often secrete peptide hormones. These tumors most commonly originate in the gastrointestinal and respiratory tracts [1,2]. Head and neck NENs are rare, accounting for less than 1% of all head and neck malignancies [3]. According to the 2022 World Health Organization (WHO) classification, neuroendocrine neoplasms (NENs) are classified into two main categories on the basis of differentiation: well-differentiated neuroendocrine tumors (NETs) and poorly differentiated neuroendocrine carcinomas (NECs) [4]. Head and neck neuroendocrine carcinomas (HN-NECs) are highly aggressive malignancies with high rates of local and distant failure, leading to poor survival outcomes. Among all histological subtypes, SCNEC shows the worst prognosis [5]. As a rare head and neck malignancy, HN-NEC often presents diagnostic challenges, frequently necessitating secondary diagnoses and even reclassification between benign and malignant tumors [6].

In clinical practice, treatment strategies for HN-NEC are often extrapolated from experience with NENs originating in other sites, such as the lung or gastrointestinal tract [7,8]. Treatment modalities include surgery, radiotherapy, chemotherapy, and various combinations of these modalities. Multimodality treatment centered on chemoradiotherapy, rather than surgery alone, is frequently recommended in previous studies based on large-scale databases, systematic reviews, and meta-analyses [9]. However, there are currently no standardized global treatment guidelines for HN-NEC. Prognostic factors also remain poorly defined and controversial [10,11]. This retrospective cohort study aims to evaluate the respective roles of radiotherapy, surgery, and chemotherapy in managing HN-NEC. It also seeks to characterize the clinicopathological features of this disease and identify critical prognostic factors to provide an evidence-based foundation for developing more effective, individualized treatment strategies.

## 2. Materials and Methods

### 2.1. Study Design

This retrospective study enrolled 39 patients and collected clinical-pathological data of patients pathologically diagnosed with poorly differentiated HN-NECs between October 2006 and May 2025 in the electronic medical record database of West China Hospital, Sichuan University. Collected variables included age, gender, primary tumor site, TNM stage, Ki-67 index, and Epstein–Barr virus-encoded small RNA (EBER) status.

Tumor staging was performed according to the American Joint Committee on Cancer (AJCC), 8th edition. As no dedicated TNM staging system exists for HN-NECs, we uniformly applied the standard AJCC 8th edition’s criteria for head and neck squamous cell carcinoma from the same anatomic sites. This approach follows current clinical practice and previously published studies on HN-NEC [12,13]. All staging was retrospectively reviewed and confirmed.

Patients were included if they had a histopathologically and immunohistochemically confirmed diagnosis of HN-NEC, with a documented primary tumor originating from head and neck regions such as the larynx, hypopharynx, nasopharynx, or sinonasal tract. Additionally, complete treatment data were required. Exclusion criteria comprised an unclear pathological diagnosis, neuroendocrine tumors metastatic to the head and neck, the presence of other concurrent malignancies that could affect survival outcomes, and insufficient essential clinical information such as treatment details.

This study was approved by the ethics committee of the cancer center of West China Hospital, with waiver of consent documentation (2025-2376).

### 2.2. Statistical Analysis

The Kaplan–Meier (KM) method was used to analyze Overall survival (OS), Locoregional recurrence-free survival (LRRFS), and Distant metastasis-free survival (DMFS). Survival rates at five years were estimated through Kaplan–Meier analysis. Survival differences between groups were compared using the log-rank test. All events were measured from the date of diagnosis to the first documented occurrence of the respective event. OS was defined as the time from diagnosis to death from any cause. LRRFS was defined as the time from diagnosis to locoregional failure or death, whichever occurred first. DMFS was defined as the time from diagnosis to distant metastasis or death, whichever occurred first. Given the retrospective nature of this study, missing data are present in Table 1. A comparison of the baseline characteristics between patients with complete and those with missing data for staging variables is provided in Supplementary Table S3. For comparative and survival analyses, only patients with complete data for the relevant variable were included. Due to the limited sample size (*n* = 39), conventional Cox regression may produce biased estimates and convergence issues. Firth’s penalized likelihood method was therefore used to reduce small-sample bias and ensure the model’s stability. Variables that were statistically significant in univariate log-rank tests were included in a multivariable Firth’s penalized Cox regression model to identify prognostic factors, reported as hazard ratios (HRs) with 95% confidence intervals (CIs). To assess the robustness of the multivariable model, we performed sensitivity analyses using alternative covariate sets. All sensitivity analyses were conducted using Firth’s penalized Cox regression. For subgroup comparisons across multiple survival endpoints, all reported *p*-values are unadjusted, and no multiplicity adjustment was applied. Therefore, these results should be interpreted as exploratory. All tests were two-sided, and the statistical significance level was set at <0.05. All statistical analyses were performed using R software (version 4.4.1).

## 3. Results

### 3.1. Patient Characteristics

Of the 68 patients initially screened, 29 (42.6%) did not meet the eligibility criteria and were excluded. The remaining 39 patients (57.4%) met the eligibility criteria and were enrolled in the study. During follow-up, 10 patients (25.6%) were lost to follow-up and were censored at their last known contact date. The final analysis included all 39 enrolled patients (Table 1). A detailed summary of individual patients’ characteristics, including treatment and outcome data, is provided in Supplementary Table S2.

The median follow-up time was 56.0 months (95% CI: 52.0–88.0). All patients whose age is over 50 years old account for (21/39, 53.8%) and the majority were male (33/39, 84.6%). Primary tumor site was stratified by larynx (18/39, 46.2%) and non-larynx (21/39, 53.8%), with the non-larynx group comprising tumors of the hypopharynx, nasopharynx, and sinonasal tract. Patients with T3 or T4 stage accounted for 43.6%. In our cohort, only 4 of the 20 patients tested for EBER were positive, and all positive cases occurred in the nasopharynx.

The therapeutic strategy for these patients incorporated surgery alone (*n* = 10), concurrent chemoradiotherapy (CCRT; *n* = 10), surgery with CCRT (*n* = 10), surgery with radiotherapy (*n* = 7), and surgery with chemotherapy (*n* = 2). Radiotherapy was delivered using intensity-modulated radiotherapy (IMRT), with doses ranging from 40 to 60 Gy in 20 to 30 fractions. Chemotherapy regimens were primarily Etoposide plus Cisplatin (EP) or Paclitaxel plus Platinum (TP), with three patients receiving cisplatin alone.

### 3.2. Survival Analysis

**Figure 1:**
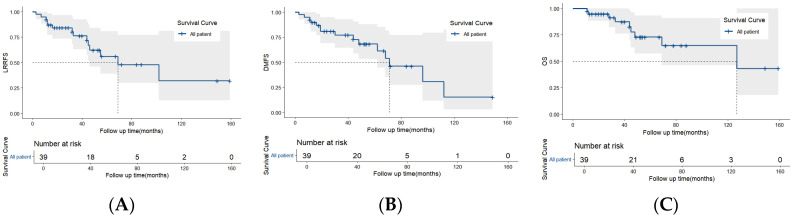
Kaplan–Meier curves for all patients of (**A**) LRRFS, (**B**) DMFS, and (**C**) OS. The grey band represents the 95% confidence interval. Abbreviations: LRRFS, locoregional recurrence-free survival; DMFS, distant metastasis-free survival; OS, overall survival.

As of the cut-off date (1 June 2025), the 5-year LRRFS, DMFS, and OS rates were 53.1% (95% CI: 32.7–73.5), 66.0% (95% CI: 48.5–83.6), and 71.6% (95% CI: 53.4–89.8), respectively (Figure 1).

**Figure 2:**
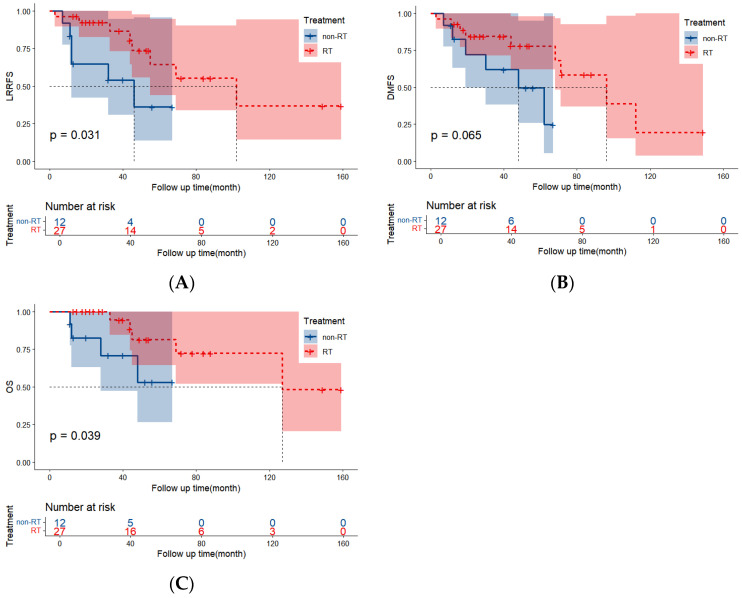
Kaplan–Meier curves for patients stratified by treatment including performing radiotherapy for (**A**) LRRFS, (**B**) DMFS, and (**C**) OS. Abbreviations: RT, the group who have undergone radiotherapy in the initial therapy; non-RT, the group who did not undergo radiotherapy in the initial therapy.

**Figure 3:**
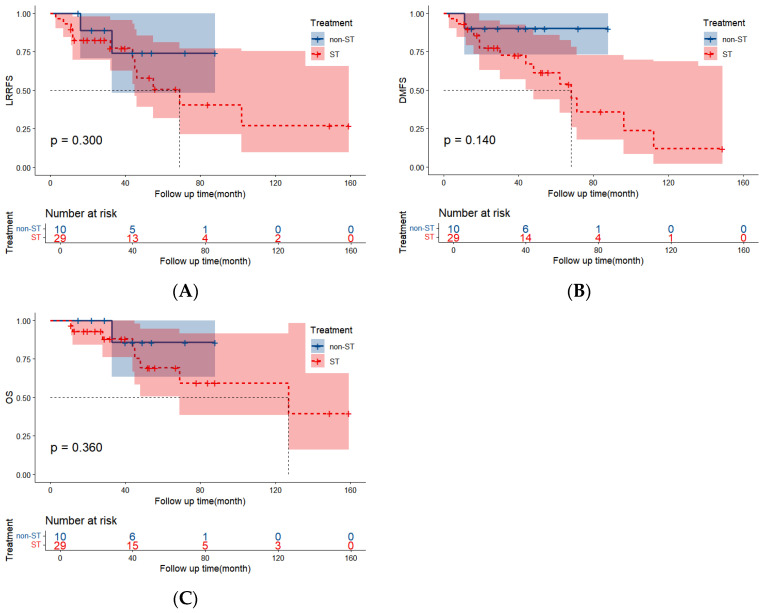
Kaplan–Meier curves for patients stratified by treatment, including performing surgery for (**A**) LRRFS, (**B**) DMFS, and (**C**) OS. Abbreviations: ST, the group who have undergone surgery in the initial therapy; non-ST, the group who did not undergo surgery in the initial therapy.

**Figure 4:**
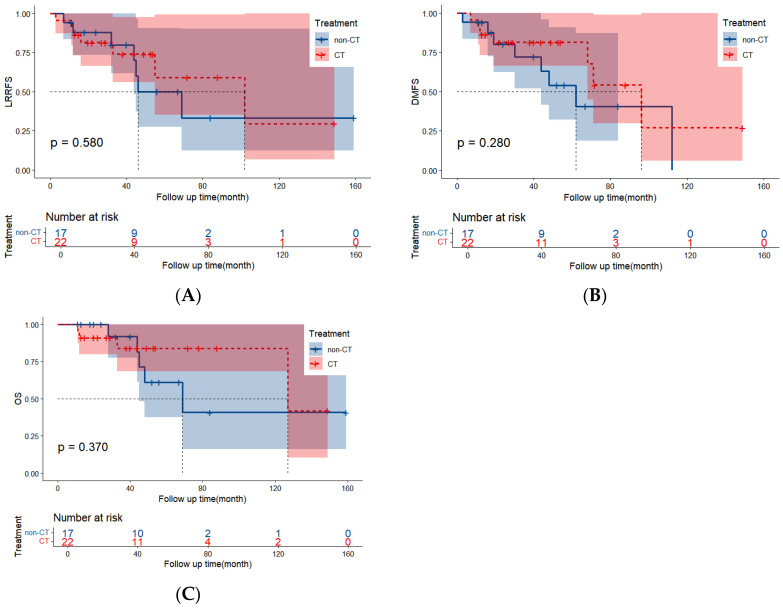
Kaplan–Meier curves for patients stratified by treatment including performing chemotherapy for (**A**) LRRFS, (**B**) DMFS, and (**C**) OS. Abbreviations: CT, the group who did not receive chemotherapy in the initial therapy; non-CT, the group who did not receive chemotherapy in the initial therapy.

Survival analysis revealed that radiotherapy reduced the local recurrence rate and improved OS. Patients receiving radiotherapy had superior 5-year LRRFS (63.2% versus 29.6%; *p* = 0.031) and OS (81.4% versus 46.9%; *p* = 0.039), with a trend toward better DMFS (75.5% versus 48.0%; *p* = 0.065) (Figure 2A,C). The 5-year LRRFS, DMFS, and OS rates for patients who did and did not receive surgery were 47.3% vs. 72.9% (*p* = 0.300), 59.5% vs. 87.5% (*p* = 0.140), and 67.1% vs. 85.7% (*p* = 0.360), respectively. Patients who received chemotherapy showed a non-significant trend toward improved 5-year DMFS (78.9% versus 51.9%; *p* = 0.280) and OS (82.1% versus 60.6%; *p* = 0.370), with LRRFS showing no marked difference (56.6% versus 46.9%; *p* = 0.580). No significant difference in survival outcomes was observed between the surgery and non-surgery groups, or between the chemotherapy and non-chemotherapy groups (Figure 3 and Figure 4).

### 3.3. Prognosis Analysis

Univariate analysis identified three factors associated with poorer OS: primary tumor site in the larynx (*p* = 0.027), age > 50 years (*p* = 0.006), and lymph node metastasis (*p* = 0.023). Two factors were associated with poorer DMFS: primary tumor site in the larynx (*p* = 0.023) and age > 50 years (*p* = 0.008). Four factors were associated with poorer LRRFS: T3-4 stage (*p* = 0.019), age > 50 years (*p* = 0.008), lymph node metastasis (*p* = 0.048), and clinical Stage III–IV (*p* = 0.013) (Table 2). Patients with missing data were excluded from the corresponding analyses.

For multivariable analysis, variables were selected based on a combination of statistical significance (*p* < 0.05 in univariable log-rank tests) and clinical relevance. Due to the limited number of events and the risk of model overfitting, the final multivariable model included age, primary tumor site, radiotherapy, and clinical stage. Although T stage and N stage were significant in univariable testing, they were excluded to avoid model overfitting and instability. Multivariable Firth’s penalized Cox regression analysis indicated that age (HR = 5.350, 95% CI: 1.120–34.482, *p* = 0.035), primary tumor site (HR = 4.513, 95% CI: 1.016–21.655, *p* = 0.048), radiotherapy (HR = 0.152, 95% CI: 0.025–0.757, *p* = 0.022), and clinical stage (HR = 9.052, 95% CI: 2.214–3072.480, *p* = 0.004) were associated with LRRFS. Clinical stage (HR = 8.215, 95% CI: 1.134–1168.863, *p* = 0.034) was associated with DMFS (Table 3). The association between radiotherapy and improved LRRFS remained consistent across all sensitivity analyses (HR range: 0.152–0.183, all *p* < 0.05), indicating that the primary multivariable results are robust (Supplementary Table S4). Given the small sample size and low number of events, the precision of several hazard ratio estimates is limited. These results should be interpreted as exploratory and require validation in larger cohorts.

### 3.4. Failure Patterns

**Figure 5:**
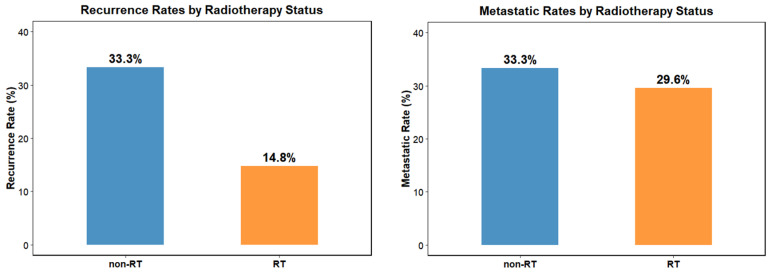
Failure pattern in the RT group and non-RT group.

Among the 39 patients, 15 patients experienced recurrence or metastasis. Specifically, three patients developed isolated local recurrence, seven patients developed isolated distant metastasis, and five patients developed both local recurrence and distant metastasis (as shown in Supplementary Table S1). A total of nine patients had died. The local recurrence rates were 14.8% in the radiotherapy group versus 33.3% in the non-radiotherapy group (*p* = 0.834), while the distant metastasis rates were 29.6% versus 33.3% (*p* = 0.148), respectively (Figure 5). No statistically significant differences were observed in the patterns of treatment failure between the two groups.

## 4. Discussion

In our study, most patients were male (84.6%). The larynx was the most common primary site (46.2%). These findings are in line with previous reports [14,15]. At initial diagnosis, nearly half of the patients (46.2%) presented with advanced-stage disease (Stage III/IV). Only 17.9% had early-stage disease (Stage I/II). This distribution indicates the aggressive behavior of HN-NEC and the imperative to identify molecular markers that could facilitate earlier intervention. EBER positivity was rare in our cohort. This observation is consistent with other published studies [16,17].

Recent research indicates that diagnostic discrepancy rates for rare head and neck tumors can reach 15% [6]. In clinical practice, seeking a second opinion from expert pathologists is common for head and neck histology. When treatment outcomes differ notably from what the initial diagnosis would predict, further immunohistochemical or molecular testing is often needed to confirm the diagnosis. Olfactory neuroblastoma (ONB) and NEC share a neuroendocrine phenotype. CK8/18 immunostaining helps distinguish between them. For sinonasal tumors with neuroendocrine features, including CK8/18 in the pathological workup is strongly recommended [18]. Diffuse insulinoma-associated protein 1 (INSM1) expression strongly supports the diagnosis of NENs [4]. HN-NEC frequently exhibits p16 overexpression and a high Ki-67 proliferation index, often exceeding 55% [19,20]. Loss of nuclear retinoblastoma protein (Rb) expression and aberrant p53 expression are hallmark features of NECs [4]. In contrast, somatostatin receptors (SSTRs) are highly expressed in NETs but not in NECs [4,21,22,23].

Squamous cell carcinoma (SCC) is the most common tumor type in the head and neck. In laryngeal and hypopharyngeal SCC, laryngopharyngeal surgery and primary chemo-/radiation (p C/RT) are well-established treatment strategies for locally advanced disease. Compared with p C/RT, laryngeal preservation surgery (LPS) is associated with significantly better overall survival in cT1-2N+M0 patients, while initial laryngopharyngeal surgery (with or without radiotherapy) provides superior 5-year overall survival and locoregional control in cT3-4N0/+M0 disease [24,25,26,27,28]. In contrast, for HN-NEC, radiotherapy appears to be the primary modality for local control and improved survival. The role of laryngeal preservation surgery in laryngeal NEC remains unclear. In head and neck SCC, nodal positivity significantly reduces overall survival, and a higher lymph node ratio (LNR) is associated with worse survival [29]. Among patients with cN0 disease, the rate of occult nodal metastases ranges from 2% to 58% in the literature [30]. Compared with neck irradiation, neck dissection offers the advantage of detecting occult metastases, enabling timely intervention. However, neck dissection is associated with higher surgical morbidity. In locally advanced laryngeal SCC without midline involvement, contralateral elective neck dissection may be omitted to reduce operative time and morbidity, highlighting the value of selective neck dissection strategies [31]. In HN-NEC, nodal metastasis is a potential prognostic factor, but the optimal extent of nodal intervention remains undefined. These findings highlight that SCC treatment paradigms cannot be directly applied to HN-NEC, underscoring the need for histology-specific strategies.

LCNEC may confer a better prognosis than SCNEC [32]. However, treatment strategies for both poorly differentiated subtypes often overlap, with a preference for systemic therapy combined with radiotherapy [33]. A pooled analysis of 701 published cases restricted to sinonasal neuroendocrine carcinomas identified differentiation grade as the most important prognostic factor, with treatment modality also playing a significant role in survival outcomes [10]. The role of surgery remains controversial. Some meta-analyses have suggested that surgery should serve as the cornerstone of treatment. Some research advocates surgery for sinonasal SCNEC or early-stage disease [10,14,34]. In contrast, our study found no survival benefit from adding surgery to chemoradiation in HN-NEC. This finding is consistent with analyses from the National Cancer Database (NCDB) and the Surveillance, Epidemiology, and End Results (SEER) program [13,35,36]. A recent systematic review further emphasized that the therapeutic value of surgery is significantly greater for SCNEC than for LCNEC. In contrast, no significant difference in radiotherapy response was observed between these two subtypes [32]. Radiotherapy emerged as a critical treatment option for local control [13,15,35]. Our univariate analysis indicates that radiotherapy may be associated with improved LRRFS and OS. Our exploratory multivariate analysis suggests a potential beneficial role of radiotherapy on LRRFS rather than on OS in patients with HN-NEC. Several explanations are possible. First, the predominant mode of death in HN-NEC appears to be distant metastasis rather than locoregional progression [13]. Therefore, improving local control alone may have a limited impact on OS, which is consistent with our finding that radiotherapy did not improve DMFS. Second, our study was underpowered to detect modest differences in DMFS or OS due to the limited sample size and events. Patients in the radiotherapy group had a lower locoregional recurrence rate than those in the non-radiotherapy group (14.8% vs. 33.3%) (Figure 5). No significant differences in treatment failure patterns were observed between the two groups, potentially due to the limited sample size. Therapeutic decision-making in HN-NEC should be guided by clinicopathological stage and differentiation grade. Current evidence suggests that NETs and early-stage (T1-2, N0-1) NECs may be managed with localized therapy. All other aggressive cases warrant multimodal treatment [9]. A stage-based treatment paradigm is increasingly recognized. Concurrent chemoradiation is recommended for Stage ≤ IVB disease, whereas systemic therapy alone is advised for Stage IVC patients [7,35]. The stage-specific treatment recommendations should be interpreted with caution, as the available evidence is derived predominantly from studies on SCNEC and may not fully reflect the biological heterogeneity of HN-NECs. Moreover, the absence of a dedicated staging system for HN-NEC further limits the applicability of these recommendations to HN-NECs. Although the optimal treatment combination remains unclear, radiotherapy appears to be associated with improved LRRFS in patients with HN-NEC in our cohort. According to available evidence, multimodal treatment incorporating radiotherapy may be considered for HN-NEC.

Numerous prognostic factors for HN-NEC have been reported in the literature. These include clinical parameters such as age, TNM stage, treatment modality and primary tumor site; pathological features including histologic subtype and grade; and molecular markers such as Ki-67 index, EBV infection, fluorodeoxyglucose (FDG) uptake, and human papillomavirus (HPV) status [10,11,15,18,19,20,35,36,37,38]. However, some studies have found that Ki-67, HPV, and EBV status showed no significant prognostic value in HN-NEC patients [9,19]. Existing evidence on the prognostic significance of NEC subtypes in the head and neck region suggests that LCNEC may confer a more favorable prognosis than SCNEC [3,30]. Our analysis identified several factors associated with survival outcomes, including age, T stage, N stage, clinical stage, primary tumor site, and radiotherapy. Patients older than 50 years exhibited inferior outcomes. The presence of nodal metastasis was associated with poorer LRRFS and OS in univariate analysis, consistent with large database studies such as NCDB and SEER [15,35]. T1-2 and clinical Stage I–II were associated with more favorable LRRFS. Laryngeal NEC was associated with a poorer prognosis compared with NEC originating from other head and neck sites. Consistent with prior literature, laryngeal NECs had the worst survival, whereas sinonasal NECs were associated with more favorable outcomes [10,35]. The prognosis for laryngeal SCNEC is particularly poor, with a 5-year overall survival rate of only 5% following chemoradiotherapy [5]. The observed poorer survival among patients with laryngeal NEC may be explained by a higher propensity for distant metastasis compared with other sites of HN-NEC [32,39,40,41]. Laryngeal NEC tends to disseminate systemically to soft tissue, lungs, and the liver (as shown in Supplementary Table S1). The observed metastatic tendency may be attributable to the distinct vascular and lymphatic anatomy of each primary site. Our exploratory analysis suggests that radiotherapy may be associated with improved locoregional control and prolonged survival. These findings indicate a potential role for radiotherapy in the treatment of HN-NEC, warranting further investigation. Factors showing significant survival differences, such as primary tumor site, age, T stage, N stage, and clinical stage may serve as a basis for subgroup analysis in future studies to better control for confounding and assess treatment effects.

Several limitations warrant consideration. This single-center retrospective study is subject to potential selection and temporal biases due to the long recruitment period and missing data. The radiotherapy regimens were heterogeneous, and subgroup analyses were not feasible due to the small sample size. Given the small sample size (*n* = 39) and the limited number of events, the confidence intervals for several hazard ratios were wide. Although Firth’s penalized Cox regression was used to reduce bias, the effect estimates should be interpreted cautiously and validated in larger cohorts. Accordingly, our findings are exploratory. Future multi-center prospective studies with larger sample sizes are warranted to validate the role of radiotherapy, to clarify subtype-specific prognostic differences, and to develop individualized treatment approaches for head and neck NEC.

## 5. Conclusions

Radiotherapy may be a protective factor and appears to contribute to local control in HN-NEC. Laryngeal NEC may be associated with a poorer prognosis. While radiotherapy was associated with favorable survival in this observational cohort, the exploratory nature of the analysis and the potential for confounding preclude definitive causal conclusions. Our results provide a foundation for future prospective studies but should not be interpreted as sufficient to guide clinical practice.

## Figures and Tables

**Table 1:** Clinical and pathological characteristics of 39 patients with head and neck neuroendocrine carcinoma.

Category	Overall (*n* = 39)
Age (%)	
≤50	18 (46.2)
>50	21 (53.8)
Gender (%)	
Male	33 (84.6)
Female	6 (15.4)
Primary tumor site (%)	
Non-larynx	21 (53.8)
Larynx	18 (46.2)
T stage (%)	
T1-2	13 (33.3)
T3-4	17 (43.6)
Unknown	9 (23.1)
N stage (%)	
N0	11 (28.2)
N1	4 (10.3)
N2	13 (33.3)
N3	1 (2.6)
Unknown	10 (25.6)
Clinical stage (%)	
I	2 (5.1)
II	5 (12.8)
III	4 (10.3)
IV	14 (35.9)
Unknown	14 (35.9)
Ki67 (%)	
<40%	9 (23.1)
≥40%	23 (59.0)
Unknown	7 (17.9)
EBER (%)	
Negative	16 (41.0)
Positive	4 (10.3)
Unknown	19 (48.7)

Abbreviations: T1-2, T stage including T1 or T2; T3-4, T stage including T3 or T4; non-larynx, including the hypopharynx, nasopharynx, and sinonasal tract.

**Table 2:** Univariate analysis of local regional recurrence-free survival, distant metastatic-free survival, and overall survival.

Factors	5-Year LRRFS (%)	*p*	5-Year DMFS (%)	*p*	5-Year OS (%)	*p*
Age		0.008 *		0.008 *		0.006 *
>50	47.3		66.6		38.1	
≤50	81.2		87.5		92.9	
Gender		0.760		0.170		0.170
Male	51.8		70.4		74.7	
Female	60		40		60	
Primary tumor site		0.150		0.023 *		0.027 *
Larynx	31.7		41.2		38.1	
Non-larynx	68.7		87.5		92.9	
T stage		0.019 *		0.200		0.150
T1-2	72.9		69.8		83.3	
T3-4	22.4		53.8		57.0	
N stage		0.048 *		0.050		0.023 *
Metastasis	27.6		45.7		44.9	
Non-metastasis	59.3		90		100	
Clinical stage		0.013 *		0.091		0.110
I–II	100		85.7		100	
III–IV	25.2		55.5		55.4	

Note: All reported *p*-values are unadjusted for multiple comparisons. These results should be interpreted as exploratory. Percentages and survival rates were calculated on the basis of these effective sample sizes. * Statistically significant association (*p* < 0.05).

**Table 3:** Multivariable Firth’s penalized Cox regression analysis of local regional recurrence-free survival, distant metastatic-free survival, and overall survival.

Factors	LRRFS		DMFS		OS	
	HR (95% CI)	*p*	HR (95% CI)	*p*	HR (95% CI)	*p*
Age						
>50/≤50	5.350 (1.120–34.482)	0.035 *	1.409 (0.332–7.452)	0.650	3.290 (0.650–31.799)	0.157
Primary tumor site						
Larynx/ non-larynx	4.513 (1.016–21.655)	0.048 *	1.079 (0.237–4.374)	0.916	0.593 (0.107–2.740)	0.500
Clinical stage						
III–IV/I–II	21.059 (2.306–2874.980)	0.003 *	9.052 (1.134–1168.863)	0.034 *	8.594 (0.890–1169.505)	0.066
RT						
Yes/no	0.152 (0.025–0.757)	0.022 *	0.543 (0.132–2.398)	0.398	0.296 (0.057–1.454)	0.128

Note: All reported *p*-values are unadjusted for multiple comparisons. These results should be interpreted as exploratory. Abbreviations: HR: hazard ratio; 95% CI: 95% confidence interval. * Statistically significant association (*p* < 0.05).

## Data Availability

The raw data supporting the conclusions of this study will be made available by the corresponding author. Data involving patient privacy is only shared upon reasonable request.

## Supplementary Materials

The following supporting information can be downloaded at: https://www.mdpi.com/article/10.3390/curroncol33070390/s1, Supplementary Table S1: Clinical Characteristics and Metastatic Patterns in Patients with Treatment Failure; Supplementary Table S2: Clinical characteristics of 39 patients with head and neck neuroendocrine carcinoma; Supplementary Table S3: Comparison of baseline characteristics between patients with and without complete T stage, N stage, or clinical stage data; Supplementary Table S4: Sensitivity analyses for the association between radiotherapy and LRRFS.

## Abbreviations

The following abbreviations are used in this manuscript.

HN-NEC	Head and neck neuroendocrine carcinoma
LRRFS	Locoregional recurrence-free survival
DMFS	Distant metastasis-free survival
OS	Overall survival
NENs	Neuroendocrine neoplasms
NETs	Well-differentiated neuroendocrine tumors
NECs	Poorly differentiated neuroendocrine carcinomas
SCNEC	Small cell neuroendocrine carcinoma
LCNEC	Large cell neuroendocrine carcinoma
EBER	Epstein–Barr virus-encoded small RNA
IMRT	Intensity-modulated radiotherapy
CCRT	Concurrent chemoradiotherapy
EP	Etoposide plus Cisplatin
TP	Paclitaxel plus Platinum
ONB	Olfactory neuroblastoma
INSM1	Insulinoma-associated protein 1
SSTR	Somatostatin receptors
SCC	Squamous cell carcinoma
LPS	Laryngeal preservation surgery
LNR	Lymph node ratio
FDG	Fluorodeoxyglucose
HPV	Human papillomavirus
