# Supplementary material for: Clinical Characteristics and Prognosis of Neuroendocrine Carcinoma in the Head and Neck: A Single-Institutional Retrospective Analysis
Source: Curr Oncol. 2026 Jun 29;33(7):390. doi: 10.3390/curroncol33070390 (PMC13409505; doi:10.3390/curroncol33070390)
Supplement: Supplementary file 1 [file curroncol-33-00390-s001.zip › Supplementary Table S1.pdf]

Clinical Characteristics and Metastatic Patterns in Patients with Treatment Failure

| No. | Age | Gender | Primary tumor site | Radiotherapy | Metastasis | Metastatic site | Recurrence |
|---|---|---|---|---|---|---|---|
| 1 | 68 | Male | Larynx | No(ST) | Yes | Pleura、Stomach、lymph nodes | Yes |
| 2 | 65 | Male | Larynx | Yes(ST+RT) | Yes | Lung | No |
| 3 | 80 | Male | Larynx | No(ST) | No | / | Yes |
| 4 | 67 | Male | Larynx | No(ST) | Yes | Stomach, Subcutaneous tissue | No |
| 5 | 66 | Female | Larynx | No(ST+CT) | Yes | Liver | No |
| 6 | 68 | Female | Larynx | Yes(ST+RT) | Yes | Neck lymph nodes, Parotid gland | No |
| 7 | 62 | Male | Larynx | Yes(ST+RT+CT) | Yes | Extensive soft tissue | No |
| 8 | 55 | Male | Larynx | No(ST) | Yes | Extensive soft tissue | Yes |
| 9 | 63 | Male | Larynx | Yes(CRT) | No | / | Yes |
| 10 | 80 | Male | Hypopharynx | No(ST) | No | / | Yes |
| 11 | 47 | Male | Nasal cavity or Paranasal sinuses | Yes(ST+RT+CT) | Yes | Brain | Yes |
| 12 | 66 | Male | Nasal cavity or Paranasal sinuses | Yes(CRT) | Yes | Frontal lobe | No |
| 13 | 28 | Male | Nasal cavity or Paranasal sinuses | Yes(ST+RT+CT) | Yes | Frontal lobe | Yes |
| 14 | 46 | Male | Nasal cavity or Paranasal sinuses | Yes(ST+RT+CT) | Yes | Supraclavicular lymph nodes | Yes |
| 15 | 50 | Female | Nasal cavity or | Yes(ST+RT) | Yes | Brain | No |

| | | |
|---|---|---|
| | Paranasal sinuses | |

Abbreviations: ST, surgical treatment; RT, radiotherapy; CT, chemotherapy; CRT, concurrent chemo-radiotherapy.
