# Supplementary material for: Clinical Characteristics and Prognosis of Neuroendocrine Carcinoma in the Head and Neck: A Single-Institutional Retrospective Analysis
Source: Curr Oncol. 2026 Jun 29;33(7):390. doi: 10.3390/curroncol33070390 (PMC13409505; doi:10.3390/curroncol33070390)
Supplement: Supplementary file 1 [file curroncol-33-00390-s001.zip › Supplementary Table S2.pdf]

Clinical characteristics of 39 patients with head and neck neuroendocrine carcinoma

ID	Sex	Age	Subtype	Primary site	Clinical stage	Treatment	Metastasis	Recurrence	Death	Survival status
1	M	58	PD-NEC	Larynx	II	ST+RT+CT	No	No	No	Alive
2	M	68	PD-NEC	Larynx	IV	ST	Yes	Yes	Yes	Dead
3	M	56	NEC-mixed	Larynx	III	ST+RT+CT	No	No	No	Alive
4	M	74	PD-NEC	Larynx	I	ST	No	No	No	Alive
5	M	56	PD-NEC	Larynx	II	ST	No	No	No	Alive
6	M	65	SCNEC	Larynx	IV	ST+RT	Yes	No	No	Alive
7	M	47	SCNEC	Larynx	IV	ST	No	No	No	Alive
8	M	64	SCNEC	Larynx	IV	ST+CT	No	NA	Yes	Dead
9	M	51	PD-NEC	Larynx	NA	ST	NA	NA	No	Lost to follow-up
10	M	80	PD-NEC	Larynx	NA	ST	No	Yes	Yes	Dead
11	M	72	LCNEC	Larynx	III	ST+RT	No	No	Yes	Dead
12	M	48	SCNEC	Larynx	NA	ST	NA	NA	No	Lost to follow-up
13	M	67	PD-NEC	Larynx	NA	ST	Yes	NA	No	Lost to follow-up
14	F	66	NEC-mixed	Larynx	IV	ST+CT	Yes	NA	Yes	Dead
15	F	68	NEC-mixed	Larynx	IV	ST+RT	Yes	NA	Yes	Dead
16	M	80	PD-NEC	Hypopharynx	III	ST	No	Yes	No	Alive
17	M	45	LCNEC	Hypopharynx	IV	ST+RT	No	No	No	Alive
18	M	68	PD-NEC	Nasopharynx	IV	RT+CT	No	No	No	Alive
19	M	41	SCNEC	Nasopharynx	NA	RT+CT	No	No	No	Alive
20	M	49	LCNEC	Nasopharynx	NA	RT+CT	No	No	No	Alive
21	F	59	SCNEC	Nasopharynx	NA	RT+CT	No	No	No	Lost to follow-up
22	M	49	LCNEC	Nasophary	NA	ST+RT	No	No	No	Lost to

ID	Sex	Age	Subtype	Primary site	Clinical stage	Treatment	Metastasis	Recurrence	Death	Survival status
				nx						follow-up
23	M	36	NEC-mixed	Nasopharynx	II	RT+CT	No	No	No	Alive
24	M	47	PD-NEC	Nasal cavity or paranasal sinuse	IV	ST+RT+CT	No	No	No	Alive
25	M	67	PD-NEC	Nasal cavity or paranasal sinuse	NA	RT+CT	NA	NA	No	Lost to follow-up
26	M	47	SCNEC	Nasal cavity or paranasal sinuse	NA	ST+RT+CT	Yes	Yes	Yes	Dead
27	M	32	SCNEC	Nasal cavity or paranasal sinuse	I	RT+CT	No	No	No	Alive
28	F	32	PD-NEC	Nasal cavity or paranasal sinuse	II	ST+RT+CT	No	No	No	Alive
29	F	29	SCNEC	Nasal cavity or paranasal sinuse	NA	RT+CT	No	No	No	Alive
30	M	66	SCNEC	Nasal cavity or paranasal sinuse	IV	RT+CT	Yes	No	Yes	Dead
31	M	28	PD-NEC	Nasal cavity or paranasal sinuse	IV	ST+RT+CT	Yes	Yes	No	Alive
32	M	34	PD-NEC	Nasal cavity or paranasal sinuse	NA	ST+RT+CT	NA	NA	No	Lost to follow-up
33	M	27	SCNEC	Nasal	NA	ST+RT	NA	No	No	Lost to

ID	Sex	Age	Subtype	Primary site	Clinical stage	Treatment	Metastasis	Recurrence	Death	Survival status
				cavity or paranasal sinuse						follow-up
34	M	41	PD-NEC	Nasal cavity or paranasal sinuse	NA	ST+RT+CT	No	No	No	Alive
35	M	46	PD-NEC	Nasal cavity or paranasal sinuse	IV	ST+RT+CT	Yes	Yes	No	Alive
36	F	50	PD-NEC	Nasal cavity or paranasal sinuse	IV	ST+RT	Yes	NA	Yes	Dead
37	M	62	PD-NEC	Larynx	II	ST+RT+CT	Yes	NA	No	Lost to follow-up
38	M	55	PD-NEC	Larynx	III	ST	Yes	Yes	No	Lost to follow-up
39	M	63	LCNEC	Larynx	IV	RT+CT	No	Yes	No	Alive

Abbreviations: F, female; M, male; HN-NEC, head and neck neuroendocrine carcinoma; NEC-mixed, mixed small cell and large cell neuroendocrine carcinoma; PD-NEC, poorly differentiated neuroendocrine carcinoma; SCNEC, small cell neuroendocrine carcinoma; LCNEC, large cell neuroendocrine carcinoma; ST, surgery; RT, radiotherapy; CT, chemotherapy.
