# Supplementary material for: Clinical Characteristics and Prognosis of Neuroendocrine Carcinoma in the Head and Neck: A Single-Institutional Retrospective Analysis
Source: Curr Oncol. 2026 Jun 29;33(7):390. doi: 10.3390/curroncol33070390 (PMC13409505; doi:10.3390/curroncol33070390)
Supplement: Supplementary file 1 [file curroncol-33-00390-s001.zip › Supplementary Table S3.pdf]

Comparison of baseline characteristics between patients with and without complete T stage data

Characteristic	T stage missing (*n*=9)	T stage complete (*n*=30)	P
Age (mean (SD))	50.89(15.41)	54.50(14.59)	0.524
Sex, female (%)	2(22.2)	4(13.3)	0.903
Primary site,Larynx(%)	1(11.1)	17(56.7)	0.043
Surgery, yes (%)	4(44.4)	25(83.3)	0.056
Radiotherapy, yes (%)	8(88.9)	19(63.3)	0.296
Chemotherapy, yes (%)	7(77.8)	15(50.0)	0.275

Comparison of baseline characteristics between patients with and without complete N stage data

Characteristic	N stage missing (*n*=10)	N stage complete (*n*=29)	P
Age (mean (SD))	52.8(15.27)	53.97(14.71)	0.832
Sex, female (%)	2(20.0)	4(13.8)	1.000
Primary site,Larynx(%)	3(30.0)	15(51.7)	0.412
Surgery, yes (%)	5(50.0)	24(82.8)	0.104
Radiotherapy, yes (%)	7(70.0)	20(69.0)	1.000
Chemotherapy, yes (%)	7(70.0)	15(51.7)	0.525

Comparison of baseline characteristics between patients with and without complete clinical stage data

Characteristic	Clinical stage missing (*n*=14)	Clinical stage complete (*n*=25)	P
Age (mean (SD))	49.21(15.08)	56.16(14.11)	0.158
Sex, female (%)	2(14.3)	4(16.0)	1.000
Primary site,Larynx(%)	4(28.6)	14(56.0)	0.189
Surgery, yes (%)	9(64.3)	20(80.0)	0.487
Radiotherapy, yes (%)	10(71.4)	17(68.0)	1.000
Chemotherapy, yes (%)	8(57.1)	14(56.0)	1.000

Footnote: Complete clinical stage data defined as availability of AJCC 8th edition stage grouping (I-IV). All reported p-values are unadjusted for multiple comparisons. These results should be interpreted as exploratory.
Abbreviations: SD, standard deviation.
