# Supplementary material for: Clinical Characteristics and Prognosis of Neuroendocrine Carcinoma in the Head and Neck: A Single-Institutional Retrospective Analysis
Source: Curr Oncol. 2026 Jun 29;33(7):390. doi: 10.3390/curroncol33070390 (PMC13409505; doi:10.3390/curroncol33070390)
Supplement: Supplementary file 1 [file curroncol-33-00390-s001.zip › Supplementary Table S4.pdf]

Sensitivity analyses for the association between radiotherapy and LRRFS

Model	Variables included	HR (95% CI) for RT	P value
Model 1	Age + primary tumor site + RT + Clinical stage	0.152(0.025-0.757)	0.022*
Model 2	Age + primary tumor site + RT + T stage	0.157(0.023-0.825)	0.029*
Model 3	Age + primary tumor site + RT + N stage	0.183(0.027-0.974)	0.046*

Model1: Age + primary tumor site + radiotherapy + clinical stage (AJCC I-IV)

Firth-penalized multivariable Cox regression analysis of local regional recurrence-free survival

Factors	HR (95%CI)	*P*
Age		
>50/≤50	5.350 (1.120-34.482)	0.035*
Primary tumor site		
Larynx/ Non-larynx	4.513 (1.016-21.655)	0.048*
Clinical stage		
III-IV/I-II	21.059 (2.306-2874.980)	0.003*
RT		
Yes/No	0.152 (0.025-0.757)	0.022*

Model2: Age + primary tumor site + radiotherapy + T stage

Firth-penalized multivariable Cox regression analysis of local regional recurrence-free survival

Factors	HR (95%CI)	*P*
Age		
>50/≤50	2.454 (0.373-16.807)	0.345
Primary tumor site		
Larynx/ Non-larynx	2.708 (0.477-13.670)	0.243
T stage		
T3-4/T1-2	10.145 (1.798-116.819)	0.006*
RT		

Factors	HR (95%CI)	*P*
Yes/No	0.157 (0.023-0.825)	0.029*

Model3: Age + primary tumor site + radiotherapy + N stage

Firth-penalized multivariable Cox regression analysis of local regional recurrence-free survival

Factors	HR (95%CI)	*P*
Age		
>50/≤50	4.692 (1.047-26.333)	0.043*
Primary tumor site		
Larynx/ Non-larynx	5.663 (1.048-34.841)	0.044*
N stage		
Metastasis/Non-metastasis	4.552 (1.109-27.262)	0.035*
RT		
Yes/No	0.183 (0.027-0.974)	0.046*

Footnote: All reported p-values are unadjusted for multiple comparisons. These results should be interpreted as exploratory.Abbreviations: RT, radiotherapy.* Statistically significant association ($p < 0.05$).
